# Conversion total hip replacement with hardware removal after open reduction and internal fixation of the acetabulum: insights from two case reports

**DOI:** 10.1093/jscr/rjaf475

**Published:** 2025-07-07

**Authors:** Nhat Dinh Vu, Tien Thanh Nguyen, Ngoc Thang Pham, Tien Thanh Pham, Dung Anh Vu

**Affiliations:** Department of Joint Surgery, 103 Military Hospital, Phung Hung street, Phuc La ward, Ha Dong district, Hanoi 12108, Vietnam; Department of Orthopaedic and Trauma, Vietnam Military Medical University, Phung Hung street, Phuc La ward, Ha Dong district, Hanoi 12108, Vietnam; Department of Joint Surgery, 108 Military Central Hospital, Tran Hung Dao street, Bach Dang ward, Hai Ba Trung district, Hanoi 11610, Vietnam; Department of Joint Surgery, 103 Military Hospital, Phung Hung street, Phuc La ward, Ha Dong district, Hanoi 12108, Vietnam; Department of Orthopaedic and Trauma, Vietnam Military Medical University, Phung Hung street, Phuc La ward, Ha Dong district, Hanoi 12108, Vietnam; Department of Joint Surgery, 103 Military Hospital, Phung Hung street, Phuc La ward, Ha Dong district, Hanoi 12108, Vietnam; Department of Orthopaedic and Trauma, Vietnam Military Medical University, Phung Hung street, Phuc La ward, Ha Dong district, Hanoi 12108, Vietnam; Department of Joint Surgery, 103 Military Hospital, Phung Hung street, Phuc La ward, Ha Dong district, Hanoi 12108, Vietnam; Department of Orthopaedic and Trauma, Vietnam Military Medical University, Phung Hung street, Phuc La ward, Ha Dong district, Hanoi 12108, Vietnam; Graduate School of Medical Science, Kanazawa Medical University, 1-1 Uchinada, Kahoku, Ishikawa 920-0293, Japan

**Keywords:** acetabular fracture, total hip replacement, hardware removal, open reduction and internal fixation, osteoarthritis

## Abstract

This case report describes two 49-year-old patients who developed post-traumatic osteoarthritis following open reduction and internal fixation (ORIF) of right acetabular fractures, necessitating hardware removal and conversion to total hip arthroplasty (THA). Using anterior approaches for ORIF and hardware removal, followed by a direct lateral approach for THA, both patients achieved significant functional improvement without complications at the 2-year follow-up. These cases highlight the challenges in managing acetabular fractures in younger patients, emphasizing the importance of tailored surgical strategies to delay THA, address bone defects, and optimize outcomes.

## Introduction

Acetabular fractures, complex injuries often caused by high-impact trauma, are influenced by hip orientation at the time of injury. These fractures frequently damage articular cartilage and increase the risk of post-traumatic osteoarthritis (PTOA) [1]. Epidemiological data show an increasing incidence, particularly among older males, with an increasing mean age at diagnosis [2]. The primary treatment goal is precise anatomical restoration of the hip to preserve mobility and reduce the risk coxarthrosis [3]. Management options include nonoperative approaches, open reduction and internal fixation (ORIF), or total hip arthroplasty (THA), but no consensus exists for complex or delayed cases [4]. PTOA, a common complication, often causes persistent pain and functional decline, with ~90% of patients requiring THA within 2 years post-fixation [5]. While THA provides significant pain relief and functional improvement, with 83% implant survival at 15 years, it is technically challenging after ORIF because of altered anatomy, bone loss, and retained hardware [6]. The management of retained hardware during THA conversion remains controversial, with limited evidence guiding decisions, especially for anterior approach fractures [7]. This case report examines two patients undergoing THA with hardware removal post-ORIF, highlighting key considerations to optimize surgical outcomes.

## Case presentation

This report presents two cases of secondary osteoarthritis following ORIF for right acetabular fractures, both requiring hardware removal and conversion to THA. These cases highlight the challenges in managing post-traumatic osteoarthritis and provide insights for optimizing surgical strategies.

### Case 1

**Figure 1:**
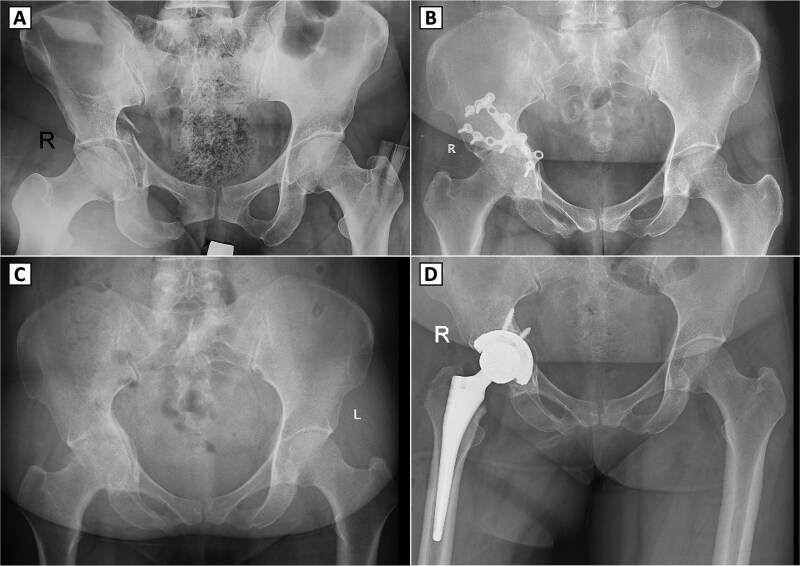
Serial radiographs of a patient with right acetabular fracture treated with open reduction and internal fixation (ORIF), followed by secondary osteoarthritis and conversion to total hip arthroplasty. (A) Preoperative anteroposterior (AP) pelvic X-ray showing a displaced right acetabular fracture. (B) Postoperative AP radiograph demonstrating internal fixation with reconstruction plates and screws on the right acetabulum. (C) Follow-up AP pelvic radiograph showing signs of secondary osteoarthritis in the right hip joint, showing shows signs of secondary osteoarthritis in the right hip joint, including joint space narrowing, femoral head deformity, and subchondral sclerosis. (D) Postoperative AP radiograph after conversion total hip arthroplasty with a cementless prosthesis and screw fixation.

A 49-year-old female sustained a right acetabular fracture from a fall and underwent ORIF via an anterior approach without immediate complications (Fig. 1A and B). The patient resumed ambulation with intermittent hip pain and did not require analgesics. At nine months post-ORIF, radiographs confirmed bony union but revealed secondary osteoarthritis (Kellgren-Lawrence grade III), with joint space narrowing, femoral head deformity, and subchondral sclerosis (Fig. 1C). Clinically, she had a limping gait, restricted hip range of motion (ROM: 98° flexion, 9° extension, 39° internal rotation, 41° external rotation, 42° abduction, and 10° adduction), yet retained squatting and leg-crossing abilities. Neurological examination and scar healing findings were unremarkable. Due to osteoarthritis progression, the hardware was removed via the anterior approach, but hip function remained unchanged. Seven months later, radiographs showed healed screw holes but advanced osteoarthritis (Kellgren-Lawrence grade IV). THA was performed via a direct lateral approach (Fig. 1D), using techniques akin to primary THA, as prior hardware removal cleared the surgical field without significant bone defects. Post-THA, immediate weight-bearing and physiotherapy were initiated, with no complications. Two years post-THA, hip function improved markedly without complications.

### Case 2

**Figure 2:**
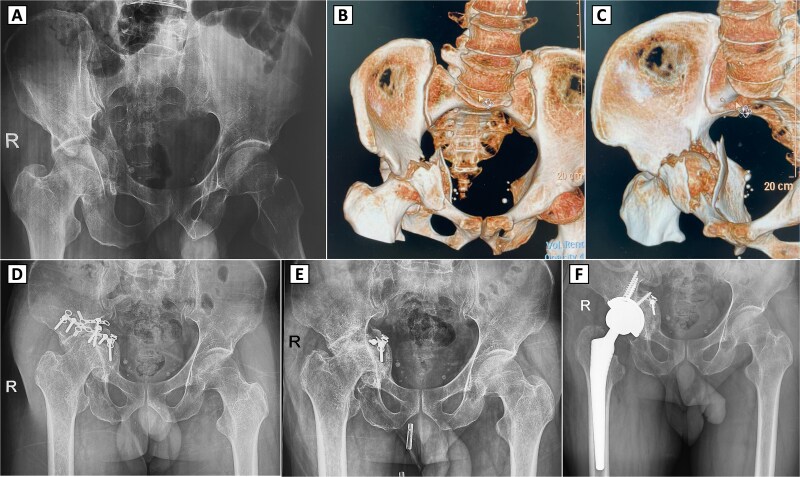
Preoperative imaging, 3D CT reconstruction, and postoperative radiographs with complex right acetabular fracture and subsequent surgical interventions. (A) Preoperative anteroposterior (AP) pelvic X-ray demonstrating a displaced right acetabular fracture with protrusion of the femoral head into the pelvis. (B, C) 3D computed tomography (CT) reconstructions (anterior and superior views, respectively) revealing the comminuted acetabular fracture involving the posterior column and posterior wall. (D) Postoperative AP pelvic radiograph showing internal fixation of the right acetabulum with reconstruction plates and screws. (E) Radiograph after hardware removal, with partial retention of fixation material, demonstrating secondary osteoarthritis of the right hip joint, including joint space narrowing and femoral head deformity. (F) Postoperative X-ray following the conversion total hip arthroplasty.

A 49-year-old male sustained a complex right acetabular fracture from a motor vehicle accident and was treated with ORIF via an anterior approach (Fig. 2A–C). The patient resumed ambulation with occasional hip pain and did not require analgesics. Seven months post-ORIF, worsening activity-related pain and a limping gait emerged, with restricted ROM (83° flexion, 7° extension, 29° internal rotation, 38° external rotation, 30° abduction, and 10° adduction). Radiographs showed partial hardware loosening, posterior femoral head displacement, joint space narrowing, femoral head deformity, and secondary osteoarthritis (Kellgren-Lawrence grade II) despite bone union (Fig. 2D). Hardware removal revealed a fractured reconstruction plate, with most components removed, except for a deeply positioned fragment deemed non-interfering for THA (Fig. 2E). Four months later, radiographs confirmed screw hole healing but progression to Kellgren-Lawrence grade IV osteoarthritis. THA via a direct lateral approach required bone grafting due to acetabular bone loss, using autogenous bone from acetabular reaming and the femoral head (Fig. 2F). Two years post-THA, hip function improved significantly without complications.

## Discussion

Acetabular fractures in younger patients pose significant challenges due to the high risk of PTOA following ORIF, often leading to THA [3]. ORIF remains the gold standard for restoring the bony anatomy in younger patients, facilitating potential future THA [8, 9]. However, non-anatomic reduction significantly predicts THA conversion by causing joint incongruity and accelerating degenerative changes [10]. In two cases of 49-year-old patients, progressive osteoarthritis developed post-ORIF, underscoring the difficulty of long-term joint preservation.

PTOA after ORIF causes chronic pain, impaired hip function, and frequent THA conversion [3]. Conversion THA is more complex than primary THA, with increased risks of infection (3.6%), aseptic loosening, dislocation, and heterotopic ossification [3, 11]. For younger patients, delaying THA is critical to extend the prosthetic joint lifespan and minimize early revision surgery, however, this must be balanced against osteoarthritis progression and quality-of-life impacts. Conversely, acute THA is increasingly preferred for patients aged over 60 years, although outcomes are less favorable than elective THA for primary osteoarthritis [10, 12].

Hardware removal was performed in both cases to manage the persistent pain and facilitate THA. This procedure is complex because of neurovascular proximity and adhesions, particularly with anterior approaches, with a complication rate of up to 20% (e.g. blood loss, implant breakage, and unresolved symptoms) [7]. Indications include peri-implant infection, hardware breakage, joint penetration, or THA preparation, although asymptomatic implant retention may be safe [7]. In the second case, partial hardware retention due to surgical challenges did not compromise THA outcomes, supporting selective retention when indicated.

Using anterior approaches for ORIF and hardware removal, followed by a direct lateral approach for THA, proved effective, with no complications at 2-year follow-up. Both patients achieved screw hole healing without requiring revision cups during THA, thereby reducing the risk of early revision. The first case utilized primary THA techniques, while the second involved bone grafting for bone loss, highlighting the need for tailored surgical strategies. These findings contribute to the limited literature on hardware removal via anterior approaches and emphasize the importance of preoperative planning. Surgeons must weigh hardware removal risks against THA facilitation, timing interventions to balance joint preservation and functional restoration in younger patients, and optimizing long-term outcomes [7, 10].
